# Prevalence and predicting factors of perceived stress among Bangladeshi university students using machine learning algorithms

**DOI:** 10.1186/s41043-021-00276-5

**Published:** 2021-11-27

**Authors:** Rumana Rois, Manik Ray, Atikur Rahman, Swapan K. Roy

**Affiliations:** 1 Department of Statistics, Jahangirnagar University, Dhaka, Bangladesh; 2 Bangladesh Breastfeeding Foundation (BBF), Institute of Public Health, Dhaka, Bangladesh

**Keywords:** Mental health, Decision tree, Random forest, Support vector machine, Feature selection, Confusion matrix, ROC, *k*-fold cross-validation

## Abstract

**Background:**

Stress-related mental health problems are one of the most common causes of the burden in university students worldwide. Many studies have been conducted to predict the prevalence of stress among university students, however most of these analyses were predominantly performed using the basic logistic regression (LR) model. As an alternative, we used the advanced machine learning (ML) approaches for detecting significant risk factors and to predict the prevalence of stress among Bangladeshi university students.

**Methods:**

This prevalence study surveyed 355 students from twenty-eight different Bangladeshi universities using questions concerning anthropometric measurements, academic, lifestyles, and health-related information, which referred to the perceived stress status of the respondents (yes or no). Boruta algorithm was used in determining the significant prognostic factors of the prevalence of stress. Prediction models were built using decision tree (DT), random forest (RF), support vector machine (SVM), and LR, and their performances were evaluated using parameters of confusion matrix, receiver operating characteristics (ROC) curves, and *k*-fold cross-validation techniques.

**Results:**

One-third of university students reported stress within the last 12 months. Students’ pulse rate, systolic and diastolic blood pressures, sleep status, smoking status, and academic background were selected as the important features for predicting the prevalence of stress. Evaluated performance revealed that the highest performance observed from RF (accuracy = 0.8972, precision = 0.9241, sensitivity = 0.9250, specificity = 0.8148, area under the ROC curve (AUC) = 0.8715, *k*-fold accuracy = 0.8983) and the lowest from LR (accuracy = 0.7476, precision = 0.8354, sensitivity = 0.8250, specificity = 0.5185, AUC = 0.7822, *k*-fold accuracy = 07713) and SVM with polynomial kernel of degree 2 (accuracy = 0.7570, precision = 0.7975, sensitivity = 0.8630, specificity = 0.5294, AUC = 0.7717, *k*-fold accuracy = 0.7855). Overall, the RF model performs better and authentically predicted stress compared with other ML techniques, including individual and interaction effects of predictors.

**Conclusion:**

The machine learning framework can be detected the significant prognostic factors and predicted this psychological problem more accurately, thereby helping the policy-makers, stakeholders, and families to understand and prevent this serious crisis by improving policy-making strategies, mental health promotion, and establishing effective university counseling services.

## Introduction

Stress is not a psychiatric diagnosis, but it’s closely linked to mental health conditions including depression, anxiety, psychosis and post-traumatic stress disorder [1]. Stress can be defined as, “the inability to cope with a perceived (real or imaginary) threat to one’s mental, physical, emotional, and spiritual well-being which results in a series of physiological responses and adaptations” [2]. This threat can be either positive (eustress) such as graduation or starting a new relationship, or negative, also called distress, with examples including academic probation or not being able to pay for semester fees [3]. Students attending a university can experience both eustress and distress in the chronic (such as multiple roles and inadequate finances) or life event (such as relocation and death) forms [3]. The university days of an individual are emotionally and intellectually more demanding than almost any other stage of education [4]. At this stage, an individual faces a great deal of pressures and challenges that pose a variety of physical, social and emotional difficulties [5].

During this transitional period, students need to cope with the academic and social demands that they encounter in university studies that help in their preparation for professional careers by the acquisition of professional knowledge, transferable skills, and evidence-informed attitudes [6–9]. According to a national health college survey of National Mental Health Association, 1 in 10 college students have been diagnosed with depression [10]. The latest 2014 American College Health Association report indicated that approximately half of the students reported more than average or tremendous stress within the last 12 months [11]. Moreover, scaling up mental health services will contribute to the achievement of the Sustainable Development Goal (SDG) 3 of well-being by 2030, to reduce one-third premature mortality from non-communicable diseases through prevention and treatment and promote mental health and well-being [12].

A plethora of research has focused on study of the prevalence of mental health problems among the university population and the findings suggest that throughout the world, a substantial number of university students experience mental health problems [4, 6–9, 13–23]. In Bangladesh, there is much work in the literature regarding the prevalence of mental health problems among university students and the results emphasize that the prevalence of depression, anxiety, and stress has been reported to be as high as 54.3%, 64.8%, and 59.0%, respectively [9, 24–28].

Most stress-related studies have focused on the prediction of the prevalence of mental health problems using the logistic regression (LR) model. Prognostic modelling with LR is well-established, particularly for a dichotomous outcome. Although LR is a popular machine learning (ML) model for classification, we are interested to evaluate the performance of different ML models, including LR, to predict the prevalence of stress among Bangladeshi university students. ML in healthcare generally aims to predict some clinical outcomes on the basis of multiple predictors [29, 30]. The potential of ML in healthcare is vast, with demonstrations of ML-based tools being able to achieve human-level or above diagnostic and prognostic capabilities having been described in almost every clinical specialty [31]. The ML framework may explore more vital information on this crucial public health concern issue. Therefore, we are motivated to find the risk factors (features) and predict the prevalence of stress among Bangladeshi university students.

## Materials and methods

### Participants and procedures

We conducted a cross-sectional online-based study among university students of different universities of Bangladesh from January to March 2020, just before the COVID-19 outbreak in Bangladesh. The participants were included anonymously and voluntarily. Data were collected using convenience sampling via an online self-reported survey at the different universities throughout the country. Considering the 5% level of significance, 5% acceptable margin of error $\left( {d = 0.05} \right)$, and $\left( {p = 0.363} \right)$ based on our pilot study (as 36.3% of university students reported stress within the last 12 months in our pilot study), the desired sample size has been estimated following the Cochran’s formula:

$$n = \frac{{Z_{\alpha /2}^{2} p \left( {1 - p} \right)}}{{d^{2} }}.$$

Hence, the required sample size was $n = 355.318 \approx 355$. Therefore, data from 355 participants were collected using a well-structured google form. Therefore, there were no incomplete questionnaires from any participants. The target variable, stress, was reported according to their perception of stress with a binary response (yes = 1, no = 0). Input variables were included gender, academic year, their background (department) and university, and stress-related physical activity and lifestyle variables, such as sleep duration time, pulse rate (low = less than 60 beats per minute, normal = 60 to 100 beats per minute, high = more than 100 beats per minute), systolic blood pressure (SBP), diastolic blood pressure (DBP), body mass index (BMI), drinking, and smoking habit. Students were classified according to world health organization guidelines as underweight (i.e., BMI < 20 kg/m^2^), normal weight (i.e., 20 kg/m^2^ < BMI < 25 kg/m^2^), overweight/obese (i.e., BMI > 25 kg/m^2^) based on their BMI value [32]. For sleep duration, participants were asked to report the average duration of sleep per day as normal (6–7 h), short (< 6 h), or long (> 7 h) [27]. According to the Joint National Committee report, blood pressure (BP) categories were defined as Normotensive (normal BP) if the observed SBP was between 91 and 120 mmHg or DBP was between 61 and 80 mmHg; Prehypertensive if the observed SBP was between 121 and 139 mmHg or DBP was between 81 and 89 mmHg, and considered as Hypertensive if the observed SBP was equal to or above 140 mmHg and DBP was equal to or above 90 mmHg, and finally, Hypotension was defined as SBP being equal to or less than 90 mmHg or DBP being equal to or less than 60 mmHg [33–35].

### Ethical issues

International ethical guidelines for biomedical research involving human subjects were followed throughout the study. After approval of the research proposal, ethical permission for data collection was received from the Department of Statistics, Jahangirnagar University, Bangladesh. The participants responded anonymously to the online survey by filling up an informed consent letter in the first section of the e-questionnaire. In the consent form, all the participants were provided with information concerning the research purpose, confidentiality of information, and right to revoke the participation without prior justification.

### Statistical analyses

This study aimed to classify and predict mental stress among Bangladeshi university students and assess the risk factors of their stress using different ML classification models, e.g., decision tree (DT), random forest (RF), support vector machine (SVM), and LR. Our methodology involves accordingly data collection and pre-processing, feature (the risk factors) selection using Boruta algorithm, splitting the entire data set into training and test data sets-applying ML models in the training data set and evaluate the performance of these models on the test data set, and finally using the best performed model predict mental stress based on the entire data set. The performances were evaluated using three performance parameters from the confusion matrix such as sensitivity, specificity, and accuracy, the area under the receiver operating characteristics (ROC) curve (AUC), and the *k*-fold cross-validation. All ML models were performed using the scikit-learn module in Python programming language version 3.7.3. Only the Boruta algorithm was implemented to select the risk factors using the Boruta package in the R programming language [36].

### Boruta algorithm

Boruta algorithm was performed to extract the relevant risk factors for university students’ perceived stress from this dataset. This is a wrapper build algorithm around the RF classifier to find out the relevance and important features with respect to the outcome variable [37]. The importance measure of an attribute for all trees in the forest is obtained as the loss of accuracy of classification caused by the random permutation of attribute values between objects. Hereafter, the algorithm iteratively removes the features which are proved by a statistical test to be less relevant than random probes [37].

### Decision tree (DT)

A DT is one of the most simple and intuitive techniques in ML based on the divide and conquer paradigm [38]. A DT, whose internal nodes are tests (on input patterns) and whose leaf nodes are categories (of patterns), assigns a class number (or output) to an input pattern by filtering the pattern down through the tests in the tree [39]. Each test has mutually exclusive and exhaustive outcomes [39].

### Random forest (RF)

An RF algorithm has hyper-parameters specifying the number of trees and the maximum depth of each tree (effectively how many interactions are considered in the model), whereas the decision rules are the parameters [40]. The RF is an ensemble learning approach for classification using a large collection of de-correlated DT [41]. In this experiment, we have used 100 DT and Gini for impurity index to implement the RF algorithm in Python.

### Support vector machine (SVM)

SVMs [42, 43] are supervised learning methods that analyze data and recognize patterns. For a two-class learning task, an SVM training algorithm constructs a model or classification function that assigns new observations to one of the two classes on either side of a hyper plane, making it a non-probabilistic binary linear classifier. An SVM model uses the kernel trick to map the data into a higher-dimensional space before solving the ML task as a convex optimization problem [41–44]. New observations are then predicted to belong to a class based on which side of the partition they fall. Support vectors are the data points nearest to the hyper plane that divides the classes [41]. We examined SVM models using the polynomial kernel of degree 2 and the linear kernel for this analysis.

### Logistic regression (LR)

LR is a probabilistic statistical classification model that predicts the probability of the occurrence of an event [41]. LR models the relationship between a categorical dependent variable and a dichotomous categorical outcome or feature. It is used as a binary (multiple) model to predict binary (multiple) responses, the outcome of a categorical dependent variable, based on one or more independent variables [38]. This is an assumptions-confined model, before estimating the model all the underlying assumptions need to be fulfilled, among them predictors have to be independent of each other and having a significant association with the outcome variable are the unavoidable assumptions [45].

### Confusion matrix performance parameters

A confusion matrix provides a visual representation of actual versus predicted class accuracies [41]. To visualize the performance of the classification algorithm, it compares the predicted classification against the actual classification in the form of false positive (FP), true positive (TP), false negative (FN) and true negative (TN) information [38, 41]. Therefore, the performance parameters are:

1 $${\text{Accuracy}} = \frac{{{\text{TP}} + {\text{TN}}}}{{{\text{TP}} + {\text{TN}} + {\text{FN}} + {\text{FP}}}},$$

2 $${\text{Sensitivity}} = \frac{{{\text{TP}}}}{{{\text{TP}} + {\text{FN}}}},$$

3 $${\text{Specificity}} = \frac{{{\text{TN}}}}{{{\text{TN}} + {\text{FP}}}},$$

4 $${\text{Precision}} = \frac{{{\text{TP}}}}{{{\text{TP}} + {\text{FP}}}},$$

where accuracy is the number of data points correctly classified by the classifier, sensitivity is a measure of how well a classification algorithm classifies data points in the positive class, specificity is a measure of how well a classification algorithm classifies data points in the negative class, and precision is the number of data points correctly classified from the positive class [38, 41].

### Receiver operating characteristic (ROC) curve

ROC curves offer another useful graphical representation for classifiers operating on datasets. Fawcett [46] provided a comprehensive introduction to ROC analysis, highlighting common misconceptions. The ROC curve shows the sensitivity of the classifier by plotting the rate of true positives to the rate of false positives. If the classifier is outstanding, the true positive rate will increase, and the area under the curve (AUC) will be close to 1 [38].

### *K*-fold cross-validation

Cross-validation is a verification technique that evaluates the generalization ability of a model for an independent dataset [41]. It evaluates the performance of various prediction functions. In *k*-fold cross-validation, the training dataset is arbitrarily partitioned into *k* mutually exclusive subsamples (or folds) of equal sizes. The model is trained *k* times (or folds), where each iteration uses one of the *k* subsamples for testing (cross-validating), and the remaining (*k* −1) subsamples are applied toward training the model. The *k* results of cross-validationare averaged to estimate the accuracy as a single estimation [41]. For this small sample size, we applied threefold, fivefold, and tenfold cross-validation techniques to evaluate the performance of classifiers.

## Results

A total of 355 students have participated in this survey from 28 different universities throughout Bangladesh with the highest proportion of responses from Jahangirnagar University (56.1%), followed by the University of Dhaka (5.9%) and the University of Rajshahi (5.6%), detailed information is in the supplementary file. Among the participants, 204 were female (57.5%), 22.5% were overweight/obese, 15.8% were cigarette smokers, 8.5% were alcoholic, and 30.7% of university students reported stress within the last 12 months. The majority of the students had a normal pulse rate (76.9%), 63.4% were normal sleepers, 77.5% had normotensive BP for SBP and 76.9% had normotensive BP for DBP (Table 1). Just over half of the total sample, 62.3% (*n* = 221) were graduate students, followed by 13% (*n* = 46) were first-year university students. The sample included 37.7% (*n* = 134) undergraduate students, with 33.6% (*n* = 45) of them reported stress. Graduate students were less likely to be stressed than undergraduate students, as 29.0% (*n* = 64) of graduate students reported stress. Highest proportion of participants 51.5% (*n* = 183) were from science background, followed by 18.3% (*n* = 65) were from arts. Stressed students were more likely to be male (35.1%), medical students (40%), first-year undergraduate students (41.3%), cigarette nonsmokers (39.3%), in low pulse rate (96.5%), normal sleepers (34.7%), overweight/obese (36.3%), had hypotension (100%) or hypertensive (100%) SBP, and had hypotension (100%) DBP as shown in Table 1.

**Table 1:** Frequency distribution and relationship with stress among university students

Variables	Total 355	Stress (*n* = 109; 30.7%)		
	*n* (%)	Yes (%)	*χ*^2^	*p* value
Gender				
Female	204 (57.5)	56 (27.5)	2.386	0.131
Male	151 (42.5)	53 (35.1)		
University				
1. Jahangirnagar University	199 (56.1)	59 (29.6)	38.811	0.066
2. University of Dhaka	21 (5.9)	5 (23.8)		
…	…	…		
27. National University	2 (0.6)	1 (50.0)		
28. University of Rajshahi	20 (5.6)	7 (35.0)		
Background				
Arts	65 (18.3)	20 (30.8)	2.891	0.576
Science	183 (51.5)	50 (27.3)		
Commerce	40 (11.3)	14 (35.0)		
Medical	30 (8.5)	12 (40.0)		
Engineering	37 (10.4)	13 (35.1)		
Academic year				
1st year	46 (13.0)	19 (41.3)	3.506	0.477
2nd year	33 (9.3)	8 (24.2)		
3rd year	31 (8.7)	10 (32.3)		
4th year	24 (6.8)	8 (33.3)		
Masters	221 (62.3)	64 (29.0)		
Pulse rate				
Low	57 (16.1)	55 (96.5)	200.75	< 0.001*
Normal	273 (76.9)	32 (11.7)		
High	25 (7.0)	22 (88.0)		
Alcoholic				
Yes	30 (8.5)	9 (30.0)	0.008	0.930
No	325 (91.5)	100 (30.8)		
Smoking status				
Yes	56 (15.8)	22 (29.1)	2.301	0.129
No	299 (84.2)	22 (39.3)		
Sleep time				
Less than normal	29 (8.2)	9 (31)	5.441	0.066
Normal	225 (63.4)	78 (34.7)		
More than normal	101 (28.5)	22 (21.8)		
SBP				
Hypotension	19 (5.4)	19 (100)	84.320	< 0.001*
Normotensive	275 (77.5)	59 (21.5)		
Prehypertensive	48 (13.5)	18 (37.5)		
Hypertensive	13 (3.7)	13 (100)		
DBP				
Hypotension	13 (3.7)	13 (100)	79.554	< 0.001*
Normotensive	273 (76.9)	63 (23.1)		
Prehypertensive	45 (12.7)	11 (24.4)		
Hypertensive	24 (6.8)	22 (91.7)		
BMI				
Underweight	77 (21.7)	24 (31.2)	1.710	0.425
Normal weight	198 (55.8)	56 (28.3)		
Overweight/obese	80 (22.5)	29 (36.3)		

*Statistically significant at the 0.05 level

Table 1 also exhibits that stressed participants were significantly more likely than non-stressed participants to be in a low pulse rate (*χ*^2^ = 200.75, *p* value < 0.05), had hypotension or hypertensive SBP (*χ*^2^ = 84.320, *p* value < 0.05), and had hypotension DBP (*χ*^2^ = 79.554, *p* value < 0.05).

### Features selection

**Fig. 1:**
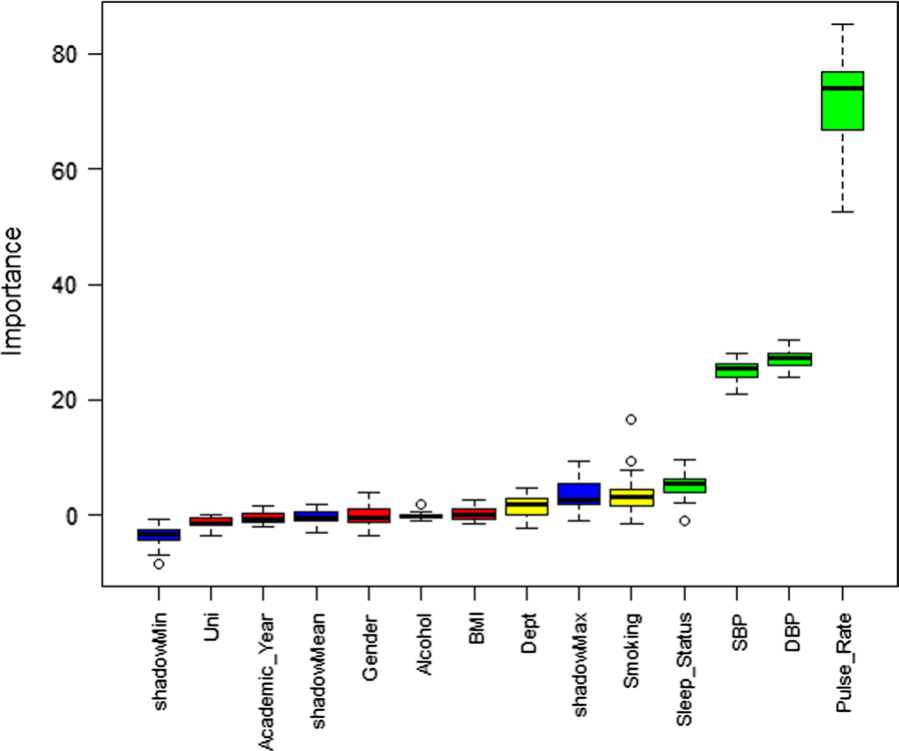
Features selection using the Boruta algorithm

Figure 1 reveals that with the aid of the Boruta algorithm, six variables (pulse rate, SBP, DBP, sleep status, smoking, background [department]) were selected among ten surveyed variables as the risk factors to predict stress among Bangladeshi university students. Students’ pulse rate, sleep status, SBP, and DBP were the confirmed features and their smoking habit and background were the tentative features for classifying their mental stress. Hereafter, these six variables were used to evaluate the performance of ML algorithms.

### Machine learning models evaluation

**Fig. 2:**
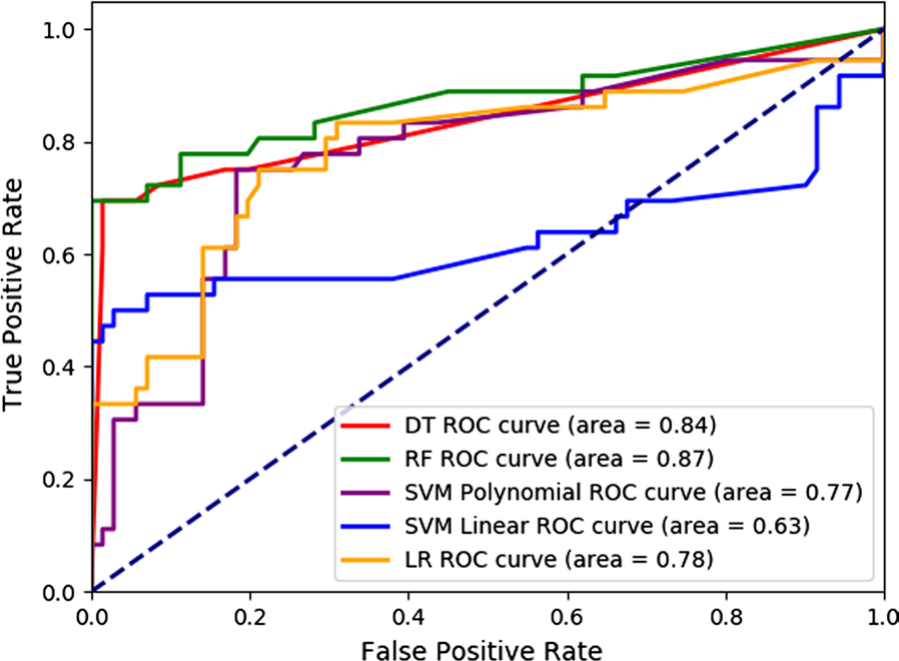
The ROC curves to predict mental stress using DT, RF, SVM, and LR models

The performance of ML models such as DT, RF, SVM, and LR were evaluated using four performance parameters of the confusion matrix (Table 2), the area under the ROC curve (Fig. 2), and the *k*-fold cross-validation approaches (Table 3). Considering 70% observations as the training data and 30% observation as the test data with the random seeds 2370–2380 for eleven different runs using the scikit-learn module, we estimated average score of accuracy, sensitivity, specificity and precision of DT, RF, SVM, and LR algorithms to predict stress among university students and the results is illustrated in Table 2. Table 2 also shows the uncertainty estimates of the parameter using the standard error (SE) of these estimated performance parameters, the standard error is the standard deviation of these estimates. The highest estimated average score of performance parameters and the lowest SE of those are indicated in bold in Tables 2 and 3, a bold value indicates a better performance of the corresponding ML model. The evaluated performances revealed that the RF model was the efficient one to predict stress among all the examined ML models based on the higher value of the estimated performance parameters and with the lower value of uncertainty of those estimates in all cases. For instance, the RF model provided 89.3% of accurate predictions (i.e., accuracy = 0.8929) with SE = 0.014, 89.5% of positive cases that were predicted as positive (i.e., sensitivity = 0.8953) with SE = 0.027, 88.5% of negative cases that were predicted as negative (i.e., specificity = 0.8853) with SE = 0.075, and 96.5% of positive predictions that were correct (i.e., precision = 0.9653) with SE = 0.021.

**Table 2:** Accuracy, sensitivity, specificity and precision of different ML models

Models	Accuracy		Sensitivity		Specificity		Precision	
	Mean	SE	Mean	SE	Mean	SE	Mean	SE
DT	0.8845	0.017	0.8908	**0.027**	0.8639	0.076	0.9581	0.024
RF	**0.8929**	**0.014**	**0.8953**	**0.027**	**0.8853**	0.075	**0.9653**	**0.021**
SVM (polynomial kernel)	0.7782	0.035	0.8504	0.047	0.6047	**0.065**	0.8406	0.039
SVM (linear kernel)	0.8054	0.039	0.8460	0.045	0.7188	0.172	0.8969	0.068
LR	0.7723	0.037	0.8160	0.045	0.6175	0.094	0.8848	0.031

*Mean* Mean of different scores of the repeated runs, *SE* Standard Error of different scores of the repeated runs

**Table 3:** Result of K-Fold cross-validation of ML Models

Models	Threefold		Fivefold		10-Fold	
	MAcc	SE	MAcc	SE	MAcc	SE
DT	0.8759	0.0419	0.8901	0.0138	0.8870	0.0361
RF	**0.8844**	0.0291	**0.8929**	**0.0126**	**0.8983**	**0.0338**
SVM (polynomial kernel)	0.7718	0.0215	0.7915	0.0559	0.7855	0.0661
SVM (linear kernel)	0.8085	**0.0072**	0.8338	0.0187	0.8309	0.0383
LR	0.7830	0.0396	0.7718	0.0566	0.7713	0.0669

*MAcc* Mean of Accuracy scores from each fold, *SE* Standard Error of Accuracy scores

Figure 2 illustrates the estimated AUC of DT, RF, SVM, and LR models, which were run using the scikit-learn module in Python 3.7.3 by considering 70% observations as training data and 30% observation as test data with the random seed 1439. To predict the prevalence of mental stress within the last 12 months among university students the estimated AUC was 0.8388, 0.8715, 0.7717, 0.6285, and 0.7822 using the ML models DT, RF, SVM with the polynomial kernel of degree 2, SVM with linear kernel, and LR, respectively. The RF algorithm performed better with the maximum AUC among all examined ML models. *K*-fold cross-validation was performed for threefold, fivefold and 10-Fold repetitions with random seed 1 and shuffle argument ‘True’, and the results is organized in Table 3. The RF model performed better in threefold, fivefold and 10-Fold cross-validations based on the higher accuracy scores, i.e., 88.4%, 89.3%, and 89.8%, respectively, and overall the lower uncertainty of the parameter estimates, i.e., 0.0291, 0.0126, and 0.0338, respectively, as shown in Table 3.

To predict the mental stress within the last 12 months among Bangladeshi university students, the RF algorithm performed better than DT, SVMs and LR algorithms based on the accuracy measure, the ROC, and the *k*-fold cross-validation approaches.

### Model to predict stress

**Fig. 3:**
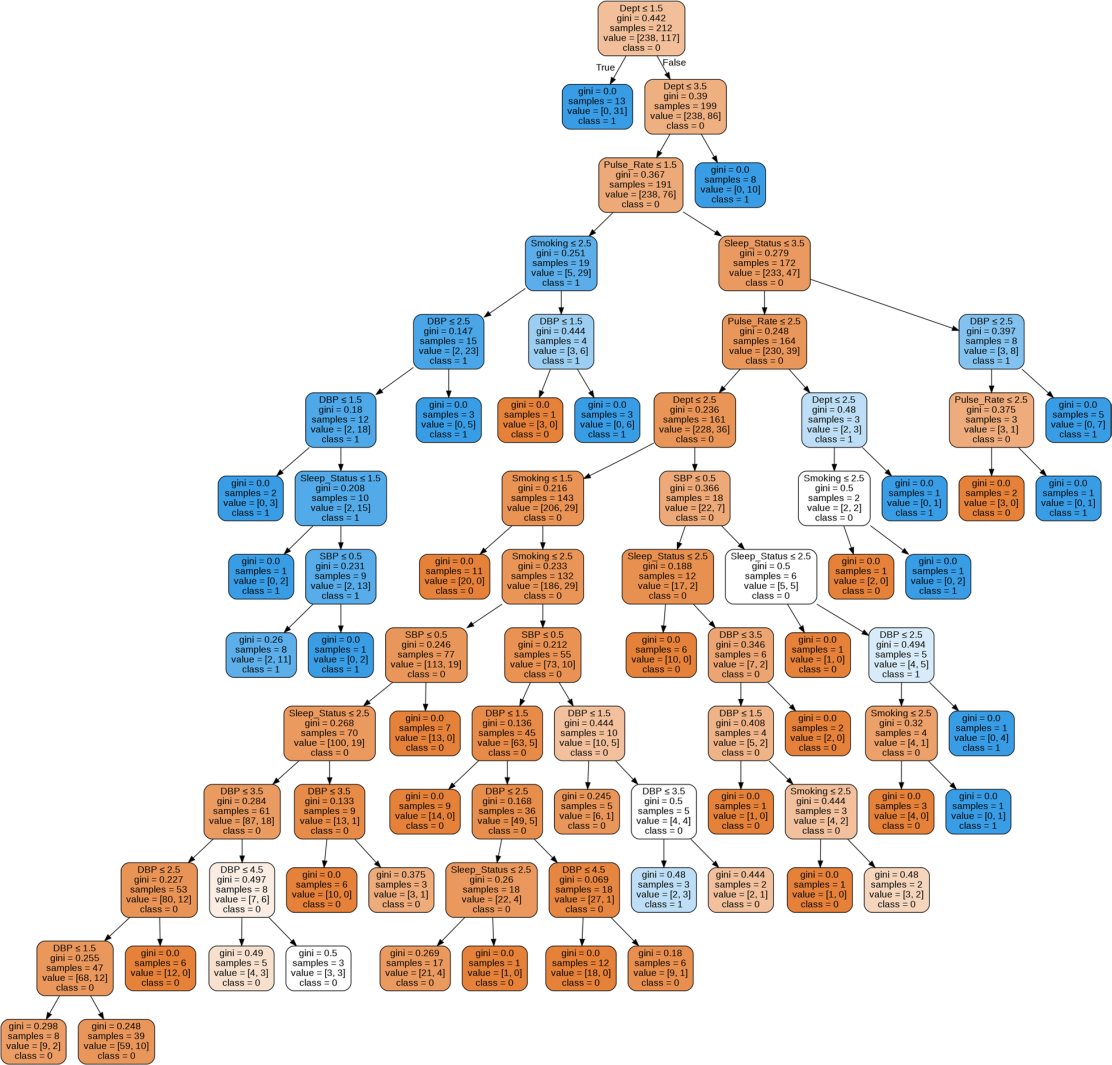
Top one tree from the fitted RF model to predict university student’s mental stress

For the entire dataset, therefore, the best performed ML model, the RF model, was fitted to predict stress using the selected significant factors—Pulse rate, SBP, DBP, Sleep status, Smoking habit, and Background (department) of students, and the top one tree from the forest is visualized in Fig. 3. All the nodes have five parts (feature’s question, gini, samples, value and class) with a question based on a value of a feature, except the terminal leaf nodes have four parts (gini, samples, value and class) [47]. The part ‘gini’ indicates the Gini Impurity of the node, which is the average weighted Gini Impurity decreases as the path move down the tree, ‘samples’ is the number of observations in the node, ‘value’ is the number of samples in each class, and ‘class’ indicates the majority classification for points in the node (‘class’ is the prediction for all samples in the leaf node) [47].

Each feature’s question has either a True (left nodes) or a False (right nodes) answer that splits the node. Based on the answer to the question, a data point moves down the tree and reaches a leaf node (the final decision). Moreover, the blue-type colored leaf indicates a prediction about stressed students and the orange-type colored leaf indicates a prediction about non-stressed students as shown in Fig. 3. To predict any given student’s data, simply move down the tree in Fig. 3, using the answer to the feature’s question until arriving at a leaf node where the class is the prediction.

Table 4 organizes this decision path for five students’ given data on their pulse rate, SBP, DBP, smoking habit, sleep status, and background (Dept) to predict their stress condition using the fitted RF model (Fig. 3).

**Table 4:** Prediction of university student’s stress using the fitted RF model

Pulse rate	SBP	DBP	Smoking	Dept	Sleep status	Predicted stress
High	Hypertensive	Hypertensive	No	Arts	Normal	Stressed
Normal	Hypotension	Hypotension	No	Science	More than normal	Non-stressed
High	Normotensive	Hypotension	No	Medical	Normal	Non-stressed
Normal	Prehypertensive	Prehypertensive	Yes	Engineering	Less than normal	Stressed
Low	Normotensive	Hypotension	Yes	Medical	Less than normal	Stressed

LR analysis further revealed that cigarette smoker students were 4.112 times more likely (OR = 4.112, 95% confidence interval (CI)  1.591–10.628, *p* value < 0.05) to be stressed than non-smokers. Respondents who had a normal pulse rate were less likely (OR = 0.002, 95% CI 0.000–0.013, *p* value < 0.05), and who had more than normal pulse rate were less likely (OR = 0.037, 95% CI 0.004–0.389, *p* value < 0.05) to be stress than those who had a low pulse rate (Table 5).

**Table 5:** Odds ratios (OR) with 95% CIs, and p-values obtained from the LR model

Variables	OR	(95% CI)	*p* value
Pulse rate			
Low (ref.)	1.000	–	–
Normal	0.002	(0.000–0.013)	< 0.001*
High	0.037	(0.004–0.389)	0.006*
Smoking			
No (ref.)	1.000	–	–
Yes	4.112	(1.591–10.628)	0.004*
Sleep time			
Less than normal (ref.)	1.000	–	–
Normal	5.244	(0.811–33.911)	0.082
More than normal	5.660	(0.808–39.650)	0.081
SBP			
Hypotension (ref.)	1.000	–	–
Normotensive	0.000	(0.000–.)	0.998
Prehypertensive	0.000	(0.000–.)	0.998
Hypertensive	0.315	(0.000–.)	1.000
DBP			
Hypotension (ref.)	1.000	–	–
Normotensive	0.000	(0.000–.)	0.998
Prehypertensive	0.000	(0.000–.)	0.999
Hypertensive	0.000	(0.000–.)	0.999
Background			
Arts (ref.)	1.000	–	–
Science	1.428	(0.456–4.474)	0.541
Commerce	0.655	(0.117–3.682)	0.631
Medical	2.925	(0.545–15.702)	0.211
Engineering	3.210	(0.814–12.665)	0.096

OR = 1 for the reference category

*Significant at 5% level

The fitted LR model in Table 5 illustrated that students’ smoking status and pulse rate were only the two significant factors to estimate the prevalence of stress and undetermined confidence intervals (CIs) for another two predictors, i.e., SBP (95% CI = 0.000, –) and DBP (95% CI = 0.000, –). However, smoking status cannot be considered in fitting the LR model, as this factor does not have a significant association with the outcome variable (Table 1). The chi-squared test in Table 1 divulged that pulse rate, SBP, and DBP were only the three significant factors for students’ stress. Moreover, students’ pulse rate, SBP, and DBP were significantly associated with each other, for instance, pulse rate was significantly associated with SBP (*χ*^2^ = 230.663, *p* value < 0.01) and DBP (*χ*^2^ = 247.583, *p* value < 0.01), and the association between students’ SBP and DBP was (*χ*^2^ = 415.105, *p* value < 0.01) also significant. Consequently, only one variable among the three significant factors, i.e., students’ pulse rate, SBP, and DBP, needs to be used to fit the LR model correctly in this analysis.

## Discussion

University students are more vulnerable to stress and other mental health issues, which can negatively impact their health and academic performance [48–50]. The global prevalence of moderate to extremely severe levels is 60.8% for depression, 73% for anxiety, and 62.4% for stress [6–8, 18–22]. As a result, public concern for the mental health of university students has been rising and their stress has become a noticeable concept in public health. Motivated by such a noticeable public health concern, this research was conducted a prevalence study to find the significant factors and prediction of stress among university students in Bangladesh using different ML models. This prevalence study showed that one-third of university students reported stress within the last 12 months.

The study results reveal that university students’ Pulse rate, SBP, DBP, Sleep status, Smoking status, and Background were the major significant factors for their stress using the ML features selection algorithm—Boruta. However, students’ pulse rate, SBP, and DBP were only the significant factors for their stress using the conventional chi-squared test. Stressed students were more likely to be medical students (two-fifth), cigarette nonsmokers (less than two-fifth), normal sleepers (more than one-third), in low pulse rate (less than one whole), had hypotension (exactly one-whole), or hypertensive (exactly one-whole) SBP, and had hypotension (exactly one-whole) DBP. Though stress and mental health differences exist between undergraduate and graduate students [51], the academic year was not a significant factor for our study. We observed that about two-fifths of the first year, followed by more than one-third of the fourth-year undergraduate students were stressed, whereas more than two-seventh graduate students were stressed. Gender was an insignificant factor for stress prediction, less than two-seventh of female students and more than one-third of male students were perceived stress within the last year. These findings of the current research have also differed from the earlier studies [4, 49, 52–54].

We evaluated the performance of ML models such as DT, RF, SVM, and LR to predict the stress of university students using four performance parameters of the confusion matrix, the AUC, and the *k*-fold cross-validation approaches. The RF model was performed better to predict stress in all the situations using eleven repeated runs with the highest mean estimates of performance parameters and overall the lowest uncertainty estimates of those parameters, i.e., 89.3% of accuracy, 96.5% of precision, 89.5% of sensitivity, 88.5% of specificity, 87.2% of AUC, more than 88% of accuracy in all the 3, 5, and 10-folds cross-validation techniques. The RF model was considered the individual and interaction effect of all the selected factors to predict the perceived stress of university students. Following the path in Fig. 3, for any individual student with the given data, their perceived stress can be predicted as shown in Table 4. On the other hand, the LR model failed to estimate the confidence interval for the two significant predictors (SBP and DBP) and illustrated significantly only two predictors, i.e., students’ smoking status (which does not have a significant association with stress) and pulse rate. This incomplete output is observed due to inappropriately estimating the LR model. As the LR model requires to fulfill all the underlying assumptions before estimating the model, among them predictors having a significant association with the outcome variable and their independence (to avoid the multicollinearity problem) are the foremost assumptions that need to fulfill. In this analysis, only one variable among students’ pulse rate, SBP and DBP will be used as a predictor variable in estimating the LR model correctly, as these variables were significantly associated (using the chi-squared test in Table 1) with stress and had a significant association between them. Hence, to overcome the multicollinearity problem only one variable should involve in estimating the LR model, otherwise, the results will be misleading. Furthermore, the RF model does not require any assumptions in estimating the model. Therefore, considering the better performance, the RF model will be better and authentic (in terms of fulfilling the assumptions) to predict the perceived stress of university students in this study.

Studies have also shown that mental health problems among university students are increasing in number as well as in severity [55]. Mental health problems can be a great source of psychological suffering and increase the risk of suicidal behaviors [6, 8, 18, 20, 56, 57]. Therefore, it is vital both to understand and then offer acceptable, effective, and accessible support for this potentially vulnerable group [58]. Counseling is the most consistently offered intervention and positive results have been demonstrated in services offering psychodynamic therapy, structured brief therapy, and integrative therapy [59, 60]. University counseling services in Australia, the UK, and the USA are reporting increases in help-seeking, with more students presenting with more severe problems [49, 61–63]. Although there is no noticeable awareness for university counseling services in Bangladesh, a reasonable number of researches carried out to address the prevalence of mental health problems among university students [9, 24–28, 64, 65], even during the COVID-19 pandemic [66–68].

The previous studies have been reported that the prevalence of stress among Bangladeshi university students as high as three-fifth of total respondents [9, 24–28]. However, our findings revealed that one-third of university students reported stress within the last 12 months. This lower prevalence rate of stress was observed as students were reported their last 12 months feeling of stress by a binary response question (Yes or No), which is one of the foremost limitations of this study. Furthermore, other major limitations are the small sample size for this type of analysis and the use of a convenience sample, so that students in the survey may not be representative of the general students population of Bangladesh. Instead of using a binary response pattern, any structured scale such as Perceived Stress Scale (PSS) or depression, anxiety, and stress scale (DASS–21) with larger and more representative samples, and utilizing the ML framework can be more informative to estimate the prevalence of stress of university students in Bangladesh.

Despite the study limitations, we feel our study has several appealing advantages in public health research. Conventional chi-square test identified only three variables (Pulse rate, SBP, and DBP) as significant factors that are likely to be a result of the student’s stress status, whereas the ML framework identified six variables (Pulse rate, SBP, DBP, Sleep status, Smoking, and Background) as significant factors for predicting stress in this analysis. Needless to say, this study introduces the application of different ML models in the prediction of university students’ stress, for instance, DT and RF, which do not require any assumptions and very easy (available) to implement in any standard software. Whereas, the popular classifier LR requires to fulfill all the underlying assumptions before estimating the model, among them predictors have to be independent of each other and having a significant association with the outcome variable are the unavoidable assumptions. Therefore, this commonly used prognostic modeling is difficult to estimate properly and improper estimation may result in some misleading information. Researchers can realize the limitations of the popular LR model for its assumptions confined feature from our study results. To implement the LR model authentically for this study, only one variable from students’ Pulse rate, SBP, and DBP needs to be used in predicting the stress of university students, then the estimated model outcomes will be correct but less informative.

Furthermore, the RF model included all these six significant variables to predict stress using their individual and interaction effects. Among these six significant factors, student’s pulse rate, SBP, and DBP are the physical consequences of their stress, smoking status is a negative stress-coping strategies, and background is a cause of their stress. Therefore, our study, though based on a small sample, finds that Bangladeshi university students’ study pressure has noticeable consequences on their physical and mental health and developed negative stress-coping strategies. University student counselling can help students identify the emotional issues caused by this study stress and explain why things get out of control. Student counselling can also protect a student from common negative stress-coping strategies by helping students notice the signs of those unhelpful coping methods early and break the harmful habits before they take a hold of their life. Considering the high accuracy in prediction, better performance, and assumptions-free feature, the RF model will be more authentic and informative using the country representative large sample with a detailed questionnaire to predict the perceived stress of university students. There is an increasing awareness of research to address the elevated risk of mental health problems in university students in Bangladesh, but a serious paucity of the health system, university counselling services, and policy of Bangladesh for supporting this potentially vulnerable group.

## Conclusion

This study provides further evidence for the finding of elevated prevalence rate of stress among Bangladeshi university students. This psychological problem is very threatening as that can affect students’ health, academic performance, and capacity to build their professional careers. Moreover, the magnitude of this problem needs to detect and understand, and hence, enable adequate and appropriate interventions for this vulnerable group. The ML framework can be detected the significant prognostic factors and predicted this psychological problem more accurately, thereby helping the policy-makers, stakeholders, and families to understand and prevent this serious crisis by improving policy-making strategies, mental health promotion, and establishing effective university counseling services.

## Data Availability

The datasets that support the findings of this study are available on request.
